# Magnetic Nanomotor-Based Maneuverable SERS Probe

**DOI:** 10.34133/2020/7962024

**Published:** 2020-06-05

**Authors:** Yong Wang, Yuhuan Liu, Yang Li, Dandan Xu, Xi Pan, Yuduo Chen, Dekai Zhou, Bo Wang, Huanhuan Feng, Xing Ma

**Affiliations:** ^1^Flexible Printed Electronic Technology Center and School of Materials Science and Engineering, Harbin Institute of Technology (Shenzhen), Shenzhen 518055, China; ^2^Shenzhen Bay Laboratory, No. 9 Duxue Road, Shenzhen 518055, China; ^3^School of Materials Engineering, Shanghai University of Engineering Science, Shanghai 201620, China; ^4^Key Laboratory of Microsystems and Microstructures Manufacturing, Harbin Institute of Technology, Harbin, Heilongjiang 150001, China

## Abstract

Surface-enhanced Raman spectroscopy (SERS) is a powerful sensing technique capable of capturing ultrasensitive fingerprint signal of analytes with extremely low concentration. However, conventional SERS probes are passive nanoparticles which are usually massively applied for biochemical sensing, lacking controllability and adaptability for precise and targeted sensing at a small scale. Herein, we report a “rod-like” magnetic nanomotor-based SERS probe (MNM-SP) that integrates a mobile and controllable platform of micro-/nanomotors with a SERS sensing technique. The “rod-like” structure is prepared by coating a thin layer of silica onto the self-assembled magnetic nanoparticles. Afterwards, SERS hotspots of silver nanoparticles (AgNPs) are decorated as detecting nanoprobes. The MNM-SPs can be navigated on-demand to avoid obstacles and target sensing sites by the guidance of an external gradient magnetic field. Through applying a rotating magnetic field, the MNM-SPs can actively rotate to efficiently stir and mix surrounding fluid and thus contact with analytes quickly for SERS sensing. Innovatively, we demonstrate the self-cleaning capability of the MNM-SPs which can be used to overcome the contamination problem of traditional single-use SERS probes. Furthermore, the MNM-SPs could precisely approach the targeted single cell and then enter into the cell by endocytosis. It is worth mentioning that by the effective mixing of intracellular biocomponents, much more informative Raman signals with improved signal-to-noise ratio can be captured after active rotation. Therefore, the demonstrated magnetically activated MNM-SPs that are endowed with SERS sensing capability pave way to the future development of smart sensing probes with maneuverability for biochemical analysis at the micro-/nanoscale.

## 1. Introduction

Micro-/nanomotors (MNMs) are miniaturized architectures or devices that can convert other forms of energy in the surrounding environment into mechanical motion in fluid [1, 2]. They can be powered by harnessing internal energy released from chemical reactions (e.g., catalytic reactions or enzymatic reactions) [3–8], obtaining kinetic energy converted from external physical fields (e.g., magnetic, acoustic, optical and electrical field) [9–13], or getting the aid from biological organisms [14–16]. Taking advantage of their movement at a small scale, MNMs have demonstrated revolutionary applications as functional micro-/nanorobots undertaking on-demand tasks such as targeted drug delivery [17–20], microsurgery [8, 21, 22], and environmental remediation [23–25]. In particular, MNM-based sensing has attracted significant attentions along with the rapid development of this field [24, 26, 27].

Based on the direct observation on the analyte-sensitive motion behavior of the MNMs, researchers developed a series of motor-based sensors. For instance, Kagan et al. proposed catalytic nanomotors for motion-based quantitative detection of silver ions that can increase the velocity of Au-Pt catalytic nanomotors [28]. And enzyme-modified nanorods were reported to analyze the concentration of the enzyme substrate of glucose by measuring the apparent diffusion coefficient [29]. Then, motion-based MNMs were used for water-quality testing by an enzyme-powered microfish [20]. Biomimetic enzyme-free microfish was also reported to sense toxins of water by the attenuation of the motors' locomotion [30, 31]. Similarly, Dong et al. used motion-based MNMs for pH sensing through velocity change due to variations in the surface tension and pH responsiveness of the gelatin material within the micromotors, respectively [32, 33]. And Patino et al. reported that urease-powered motors modified with fluorescence resonance energy transfer- (FRET-) labeled triplex DNA nanoswitch could sense environmental pH and monitor the intrinsic activity of the MNMs [34].Kong et al. employed the glucose oxidase enzyme and ferrocenemethanol shuttle system to detect the glucose in human serum based on the speed change of Mg/Pt Janus micromotors [35]. Wang et al. utilized DNA hybridization assay together with catalytic nanomotors for gene detection (e.g., DNA, bacterial ribosomal RNA) [36, 37]. Furthermore, with the assistance of a smartphone, the Zika virus was reported to be detected by motion-based MNMs via immunological detection [38]. However, the accuracy of the abovementioned MNM-based biochemical sensors is greatly influenced by many uncontrollable factors other than the target analyte that might also affect the velocity of the sensor motors.

Then, instead of monitoring the velocity of the motors to detect the analyte, the integration of a self-propulsion platform of MNMs and other sensing technologies opens up another direction for MNM-based biochemical sensing, where the motion behavior of the motors is utilized to assist the analyzing process. For instance, quite a few researchers modified fluorescence tags onto MNMs for fluorescence “OFF-ON” sensing. Esteban-Fernández de Ávila et al. integrated dye label with MNMs to sense miRNAs of a single cell in real time by “OFF-ON” fluorescence switching. Fluorescence was quenched for *π*−*π* interactions with the graphene-oxide surface and recovered after dye-labeled single-stranded DNA (ssDNA) binding with the target miRNA-21 [39]. Subsequently, Zhang et al. integrated carbon nanodots with magnetic spores to detect toxins of *Clostridium difficile* by fluorescence quenching [40]. In turn, kinds of MNM-based “ON-OFF” fluorescence detection were reported. Quantum dots decorated on MNMs selectively quenched fluorescence for targeted substance such as Hg^+^ [41] and bacterial endotoxin [42, 43]. Reduced graphene oxide and fluorophore fluoresceinamine were applied on MNMs for mycotoxins in food [44] and sarin [45] by fluorescent quenching. However, the fluorescence sensors transmit the signal mainly by the two states of “ON” or “OFF,” which can only deliver limited information. Besides, most of previous reports could only detect a single analyte. For unknown analytes, other sensing techniques need to be explored to couple with MNMs for advanced biochemical sensing.

Surface-enhanced Raman spectroscopy (SERS) is a powerful analytical technology that provides fingerprint molecular information by analyzing the Raman shift of optical scattering of molecular bonds [46–48]. Featured with ultrasensitive detection, quick analyzing time, and nondestructive analysis, SERS has been widely utilized in biological sensing, chemical analysis, and bioimaging [49–52]. Conventional SERS probes are generally noble metal-based (e.g., Au or Ag) nanostructures, mainly including substrate (e.g., SERS chips) [53, 54] or passive nanoparticles (AuNPs or AgNPs) [55–58] that can induce surface plasma resonance to enhance the Raman signal. For biochemical sensing applications, either analytes are dosed onto the SERS substrate or a large amount of SERS nanoprobes like AuNPs or AgNPs were added into the analyte sample. And the contact between SERS probes and analytes relies on the passive diffusion of either the analytes or SERS nanoprobes, which is a chaotic process lacking controllability for accurate detection at a small scale. Our previous work reported a self-propelled “match-like” one-dimensional SERS probe driven by photodecomposition of AgCl, where the phototaxis behavior of the nanomotors was utilized to enrich the SERS probes for an additional enhancement of SERS signal [31]. Novotný et al. and Han et al. reported SERS probes driven by the decomposition of H_2_O_2_ for the detection of explosives and Rhodamine 6G, respectively [59, 60]. These motor-based SERS probes performed collective behavior (phototaxis or micromixing) for SERS sensing applications. However, precise manipulating on a single nanomotor promises advancement of motor-based SERS sensors regarding the precisely targeted sensing and SERS performance enhancement. Up to now, compared with other propulsion mechanisms, magnetically powered motors have apparent advantages regarding the accuracy and adaptability of the motion control on the movement of MNMs [61–63]. In addition to the other merits of biocompatibility and tissue penetration of the magnetic field, magnetic MNMs thus hold a promising potential to be applied in a biosensing field [64–67].

Hereby, we proposed a “rod-like” magnetic nanomotor-based SERS probe (MNM-SP) with a core-shell structure composed of silica-coated Fe_3_O_4_ nanoparticles. Ag nanoparticles were grown on the surface of a silica coating layer to endow the nanomotors with SERS sensing hotspots. A single MNM-SP can approach target sites according to the predesigned route accurately and then actively rotate to enhance contact with analytes, achieving targeted and enhanced SERS sensing by remote manipulation. Meanwhile, through the “lab-on-chip” experiment, we for the first time demonstrated self-cleaning ability and reusability of the MNM-SP, which can overcome the drawback of analyte contamination for conventional single-use SERS probes. We further carried out biological sensing *in vitro*, where the MNM-SP can precisely navigate to a single targeted cell and enter into the cell by endocytosis. After activating the rotation of the MNM-SPs within an intracellular environment, significantly enhanced SERS signal with improved signal-to-noise ratio was acquired. And many extra Raman peaks attributed to biomolecules in the cytoplasm were captured as compared to nonrotating SERS probes, which might provide insightful biological knowledge for future intracellular biosensing analysis.

## 2. Results

### 2.1. Synthesis, Characterization, and SERS Sensing Capability of the MNM-SP

**Figure 1:**
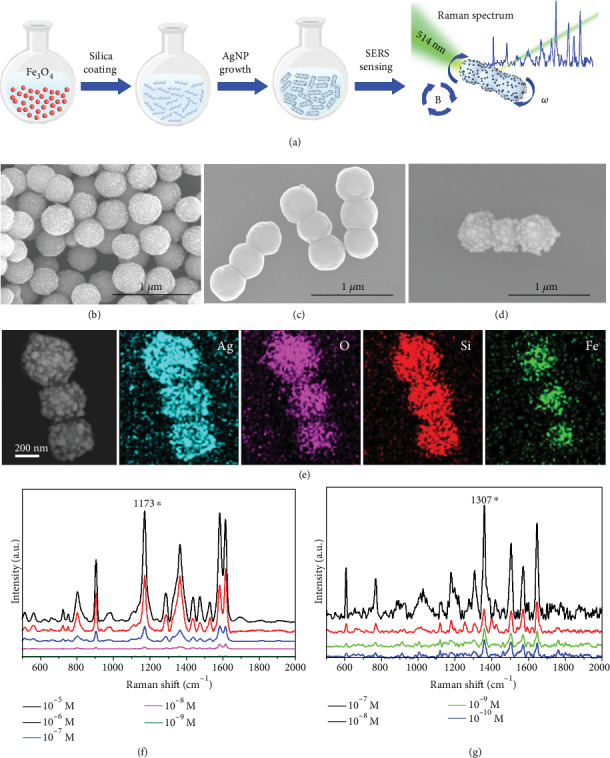
Preparation and characterization of “rod-like” magnetic nanomotor-based SERS probes (MNM-SP). (a) Schematic illustration of the fabrication and SERS sensing of the MNM-SP. SEM images of (b) Fe_3_O_4_ nanoparticles, (c) silica-coated magnetic nanorods, and (d) magnetic rod decorated with Ag nanoparticles, respectively. (e) EDS elemental mapping of “rod-like” magnetic SERS probes. Raman spectra of different concentrations of (f) CV and (g) R6G, respectively.

For obtaining magnetic nanoparticles as the main body of the “rod-like” nanomotors (Figure 1(a)), we used hydrothermal synthesis [68, 69] to acquire Fe_3_O_4_ nanoparticles with the diameter of about 400 nm (Figure 1(b)). Then, an extra magnetic field of about 0.9~3.5 mT with fixed direction was applied, the magnetic nanoparticles with permanent magnetic dipoles would align themselves according to the external magnetic field, and at the same time, also attract each other to form short chains [70] (Figure S1). Then, we grow a thin layer of silica on the external surface of the cross-linked chains to “fix” the shape of the short chains and form rigid nanorods (see more details in Materials and Methods and Figure 1(c)). And the core-shell structure is shown by the high-resolution SEM image in Figure S2. Compared with Fe_3_O_4_ nanoparticles in Figure 1(b), the surface of a nanorod (Figure 1(c)) become smooth due to the coating layer of silica. We can obtain nanorods with different lengths by tuning the assistant magnetic field strength. With the strength of assistant magnetic fields increasing from 0.9 mT to 3.5 mT, the average length of the nanorods increased from 1.3 *μ*m to 2.3 *μ*m (Figure S3).

The coating layer of silica has two functions. First, the silica layer can impose stiffness to maintain the “rod-like” structure and avoid the disassembly of the aligned cross-linked chains. Second, the silica layer can further serve as a chemically active surface allowing the growth of Ag nanoparticles by “silver mirror” reaction to obtain hotspots for SERS detection. The surface of the nanorod became rough again due to the growth of Ag nanoparticles (Figure 1(d)). The results of elemental analysis by energy-dispersive spectroscope (EDS) show that Fe and O distribute on sites of the Fe_3_O_4_ nanoparticles and Si and Ag appear throughout the whole body, suggesting successful preparation of the silica-coated “rod-like” structure decorated with Ag nanoparticles, as the MNM-SP for further study (Figure 1(e)).

Ag nanoparticles regrown on the surface of the nanorods have irregular nanostructures with sharp tips with a diameter of less than 100 nm which is very effective to excite surface plasmon for Raman signal enhancement. Besides, the gaps between the Ag nanoparticles might also provide extra SERS hotspots to further improve the SERS sensing capability [71]. To evaluate the SERS activity of the MNM-SP, typical SERS analytes, crystal violet (CV), and rhodamine 6G (R6G) were chosen as model molecules. Raman spectra with Raman shift ranging from 400 to 2000 cm^−1^ were acquired with the presence of the MNM-SP as SERS probe. The characteristic peaks of CV and R6G located at 520, 728, 804, 910, 1173, 1372, 1531, 1585, and 1617 cm^−1^ (Figure 1(f)) [72] and 610, 770, 1307, 1124, 1182, 1307, 1573, 1358, 1508, and 1647 cm^−1^(Figure 1(g)), respectively [73] were clearly presented. More details about the assignments of these characteristic peaks can be found in Table S1 and S2. We chose the prominent peaks at 1173 and 1307 cm^−1^ for CV and R6G, respectively, to evaluate the concentration-dependent SERS sensing capability of the MNM-SP (Figure S4). The MNM-SPs can detect Raman signal as low as 10^−8^ M for the CV molecule and 10^−10^ M for the R6G molecule, respectively, which is comparable to other Ag nanoparticle-based SERS probes. The good SERS performance of the MNM-SPs ensures the further motor-assisted SERS sensing applications in a follow-up study.

### 2.2. Magnetically Controlled Motion Behavior of the MNM-SPs

**Figure 2:**
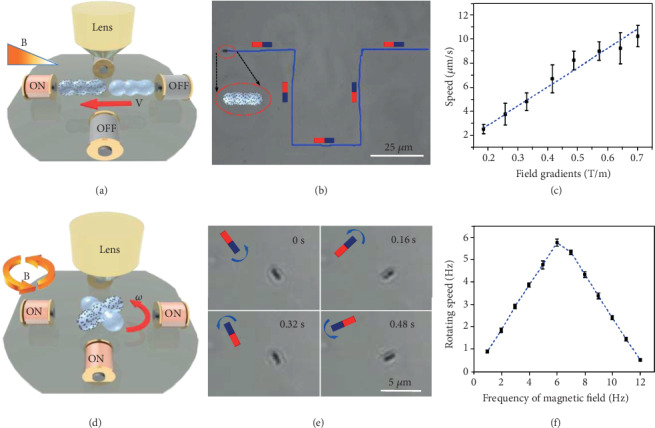
Movement of translation and rotation in deionized water. (a, d) Scheme of translational and rotational movement of the MNM-SP in gradient and rotary magnetic field, respectively. (b) Video snapshots of the MNM-SP navigating with a predesigned route by gradient magnetic field actuation. (c) Average translational speed of MNM-SPs with different magnetic field gradients. (e) Video snapshots of MNM-SPs rotating at different time intervals (frequency of the rotary magnetic field is 2 Hz). (f) Average rotating speed (1 Hz = 2*π* rad/s) (in (DI) water) of MNM-SPs in the rotary magnetic field (10 mT) with different frequencies. Error bars indicate standard deviation (*N* = 5).

The external magnetic field generated by a home-made magnetic field generator can alter the strength and orientation conveniently in real time by varying the inputting current applied on the corresponding electromagnetic coils. The MNM-SPs could be driven remotely by an external magnetic field, and the motion behavior was observed by optical microscope. When MNM-SPs were located in a gradient magnetic field, the MNM-SPs would be “pulled” by a magnetic force expressed by **F** = ▽(**m***· ***B**), where **B** is the external magnetic field and **m** is the magnetic moment (Figure 2(a)). And if the magnetic moment of the MNM-SP does not align to the orientation of the external magnetic field, a torque **τ**(**τ** = **m** × **B**) would act on the MNM-SPs and turn the MNM-SPs to line up with the direction of the external field. Meanwhile, the magnetic force would also drive the nanomotors to move towards the magnetic gradient [62–64]. (Movie S1). In this way, we can control the MNM-SPs to move along the on-demand route to avoid obstacles and approach targeted sites within a microscale and even a nanoscale (Figure 2(b)). We recorded videos and analyzed the average speed of the MNM-SPs under different magnetic field gradients (Figure 2(c)), which shows a linear relationship with the strength at the motors' site increasing up to 0.7 T/m. In the following work, we demonstrated precisely the navigation of the MNM-SP towards different sites of analytes and targeted single cell by external magnetic field, which we will discuss later.

When the MNM-SPs are located in a homogeneous magnetic field, they would not experience any magnetic force but only torque that would rotate the MNM-SPs to align with the direction of the external magnetic field as explained before. In order to rotate the MNM-SP continuously, a rotating magnetic field is employed. Signal sources were produced by a function generator and then inputted into a power amplifier to obtain an amplified current which was applied to a set of coils to produce the required gradient or rotary magnetic field. A magnetic gradient was produced by activating only a single coil of either the *X* or *Y* axis (Figure 2(a)). Sinusoidal function with a phase difference of 90° can form a rotating field whose field vector changes regularly around the *Z* axis in the *X*-*Y* plane, which can be used to rotate a rod-like MNM-SP as demonstrated in Figure S5 in the supporting information (SI). Video snapshots showing a MNM-SP rotating at a rotary magnetic field with frequency of 2 Hz are presented in Figure 2(e). The rotating frequency of the magnetic field can be altered by changing the sinusoidal frequency (Figure 2(f)). The MNM-SP experienced both the magnetic torque and the viscous resistance. Therefore, the rotating frequency of the MNM-SP cannot always keep up with the magnetic field. In our case, at a low frequency of magnetic field (<6 Hz), the torque always accelerated the rotating speed which increased from 0.9 Hz to 5.7 Hz with the rotating frequency of the magnetic field increasing. However, there is a step-out frequency that is sensitive to the viscosity of the liquid medium [74, 75]. The rotating speed of the MNM-SPs reached the maximum at 6 Hz (the step-out frequency we found here), and then, the rotating speed start to decline with magnetic field ranging from 7 to12 Hz. In addition, the MNM-SP started to oscillate and failed to follow the rotation when the rotating frequency of the magnetic field is higher than 12 Hz. (Movie S2) Besides, the MNM-SP could also be effectively activated in a high viscous environment of silicone oil (500 Cst) and reached the maximum at step-out frequency of 4 Hz. Although it is lower than the step-out frequency in (DI) water, it still proves the high efficacy of the magnetic activation of current system (Figure S6).

### 2.3. Targeted SERS Sensing and Self-Cleaning of the MNM-SPs

Generally, biochemical sensing probe-based nanomaterials, such as SERS probes, are passive nanoparticles which cannot target a local site quickly and precisely at a micro-/nanoscale [76]. And thus, usually a considerable amount of nanoprobes (nanoparticles) were applied into the sensing sample in order to achieve signals from every local site of the analyte, such as cells. The MNM-SPs presented here can be precisely navigated towards the on-demand site, which can achieve targeted sensing at a specific location. Furthermore, effective interaction between sensing probes and analytes is crucial for biochemical sensing. However, at microscale, the Reynolds number reduces rapidly and then viscous force becomes dominant and inertial force becomes negligible. Then, laminar flow often appears in a microscale environment and it is difficult and inefficient to mix a solution just by a passive diffusion of different components within the solution, which greatly limited the sensing efficacy of nanoprobes [77]. Hereby, the self-rotating MNM-SPs could effectively mix the solution to tackle this problem and increase the chance of probe-analyte contact for SERS sensing.

**Figure 3:**
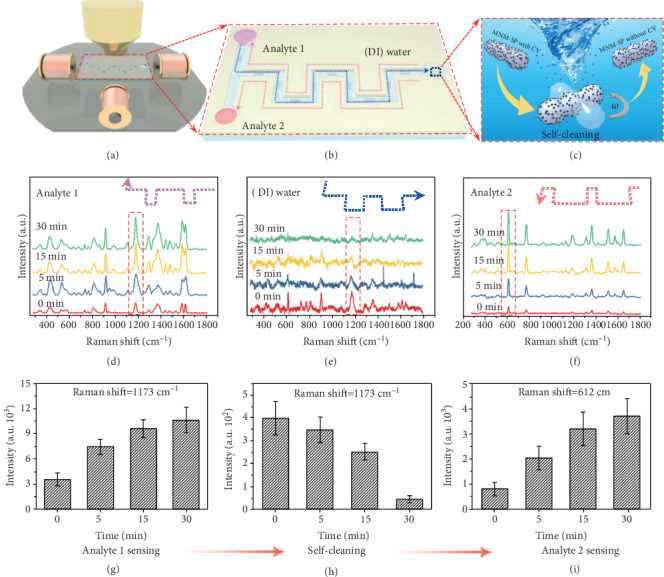
Targeted SERS sensing and self-cleaning of the MNM-SP. Schematic illustration of (a) the setup showing the on-chip experiment. (b) Schematic illustration showing manipulation of the MNM-SP self-cleaning and sensing process: the MNM-SPs moved to the tank containing CV for analyte 1 sensing (pink dotted line); then, the contaminated MNM-SPs by the CV moved to the tank with deionized (DI) water for self-cleaning (blue dotted line), and finally the cleaned MNM-SPs were reused for analyte 2 sensing (red dotted line). (c) Schematic illustration of the rotation-enabled self-cleaning of the MNM-SP. (d–f) The Raman spectra of the analyte 1 (CV) sensing, CV-contaminated probes during self-cleaning, and analyte 2(R6G) sensing, respectively, with different rotating times. (h–j) The intensity variation of the prominent peaks of CV and R6G in (d–f), respectively. Error bars indicate standard deviation (*N* = 5).

We designed and fabricated a microchannel with three tanks connected with a twisting channel (Figures 3(a) and 3(b) and Figure S7) to demonstrate the targeted SERS sensing and self-cleaning ability of the MNM-SPs. Two tanks on the left side were loaded with CV and R6G, respectively, as analytes, and the tank at the right side was filled with deionized (DI) water, where the MNM-SPs were first added and moved to a CV tank driven by the gradient magnetic field remotely (pink dotted line route). Then, a homogeneous rotary magnetic field was applied to rotate the MNM-SPs to enhance the contacts between the SERS hotspots with analytes (CV) in the solution (Figure 3(b)), then the SERS signal was collected. Characteristic peaks of analyte 1 (CV) was acquired (Figure 3(d)). For the future unknown sample, such fingerprint Raman spectra can be used to identify the analyte. More importantly, the intensity of Raman signal shows a much higher increasing trend with magnetic stir time increasing (Figure 3(d) and 3(g)) compared with the control groups without rotation (Figure S8(a) and (d)), suggesting the effectiveness of the mixing effect as mentioned before.

Meanwhile, traditional SERS probes are “contaminated” by the sample after first-time detection and thus cannot be reused for other analytes within the same circumstance. The MNM-SPs can overcome this limitation through rotation-assisted self-cleaning. Here, we recovered the CV “contaminated” MNM-SP by moving them back to the tank of DI water (blue dotted line route), where the MNM-SP could wash away the adsorbed CV molecules by magnetically driven stirring within DI water (Figure 3(c)). After stirring in DI water for 30 minutes, the Raman signal of CV adsorbed on the MNM-SP gradually decreased to be a negligible value (Figure 3(e) and 3(h)). However, without rotation, the Raman signal from the adsorbed CV was maintained (Figure S8(b) and (e)). It is anticipated that higher rotation speed and longer rotation time would enhance such self-cleaning capability. Furthermore, the strength of the interactions between the analytes and MNM-SPs, like electrostatic binding and van der Waals force, would also affect the self-cleaning efficiency. Then, the MNM-SPs were driven to the tank with analyte 2 (R6G) (red dotted line route). With the assistance of rotation, the characteristic Raman peaks of R6G was identified and obviously enhanced as well (Figures 3(f) and 3(i)). By combining with micro-/nanomotors as a controllable moving platform, the MNM-SP can approach targeted sites by magnetic navigation, which greatly improves the sensing efficacy compared to traditional SERS probe that can get in contact with analytes just by passive diffusion. Furthermore, the activated MNM-SP with stirring capability can not only increase the changes to contact with analytes for rapid detection but also recover the SERS probes by removing contaminated analytes for reusing.

### 2.4. Intracellular SERS Sensing by a Single MNM-SP

**Figure 4:**
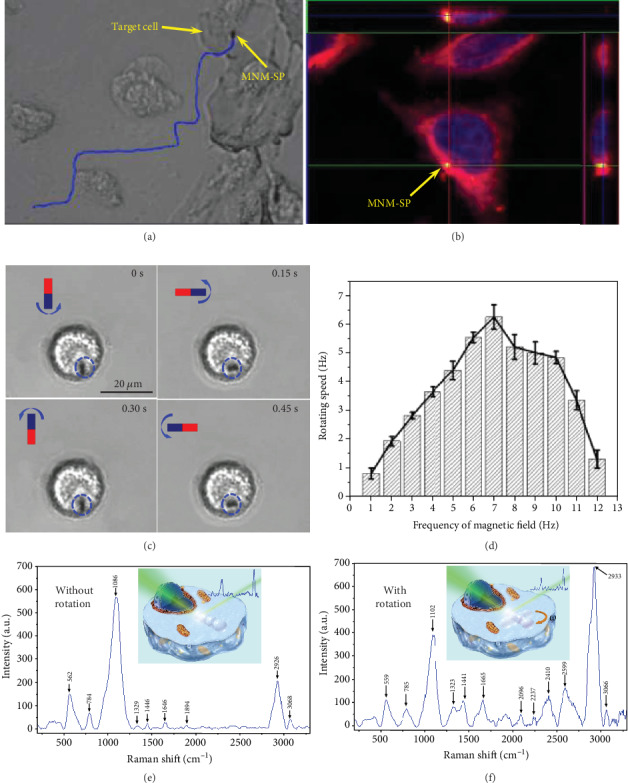
Intracellular Raman sensing by a single MNM-SP. (a) Video snapshot of an MNM-SP approaching a target HepG-2 cancer cell by magnetic navigation. (b) CLSM image of an MNM-SP (yellow) uptaken into a cell. (c) Video snapshots of an MNM-SP rotating inside a living cell at different time intervals. (frequency of the rotary magnetic field (20 mT) is 2 Hz). (d) Rotating speed of the MNM-SP in the cell with different frequencies of the rotary magnetic field (20 mT). Typical SERS spectra from the site of the MNM-SP within an intracellular environment (e) before and (f) after rotation, respectively (inserted pictures are the schematic illustration of the intracellular SERS sensing by MNM-SP without and with rotation).

For SERS sensing, the traditional SERS probes of Au or Ag nanoparticles were cocultured with cells and entered into cells by endocytosis, where the chance for probes to contact with the cell membrane is random and uncontrollable. We used a single MNM-SP as the sensing probe to approach a single targeted cell and actively contact with the cell membrane (Figure 4(a) and Movie S3). Then, the cell would start the endocytosis process to internalize the MNM-SP, in which the MNM-SP would be wrapped in a vesicle that would eventually separate from the cell membrane to enter into an intracellular environment. With the assistance of a magnetic force, we anticipate improved binding affinity between the MNM-SP and cell membrane, which should facilitate the endocytosis process. In order to track the MNM-SP inside cells, we dyed MNM-SP with green fluorescence by fluorescein isothiocyanate (FITC) (Figure S9). Then, the cell nucleus was stained with blue fluorescence dye of 4′,6-diamidino-2-phenylindole (DAPI) and the cell membrane with red fluorescence dye of 1,1′-dioctadecyl-3,3,3′,3′-tetramethylindocarbocyanine perchlorate (DIL) (see more details in Materials and Methods section). The confocal laser scanning microscope (CLSM) was employed to track and observe the MNM-SP shown by a yellow color in the cell due to the overlap between green (FITC on motor) and red colors (DIL) (Figure 4(b) and Movie S4). The cross-sectional view of the CLSM image on the right and upper sides of Figure 4(b), respectively, proved that the MNM-SP was indeed uptaken into the cell by endocytosis.

Due to the high viscosity of the cytoplasm, it is hard for SERS probes to move themselves inside the cell. Besides, micro-/nanoparticle-based SERS probes endocytosed are usually entrapped inside the endosome, an additional biological barrier, preventing the SERS probes to contact with different biomolecules outside the endosome and other subcellular components inside the cells. Here, we applied a rotating magnetic field to rotate the MNM-SP inside the cell. We expected that the rod-like MNM-SP can act as a stir bar to probably break the endosome mechanically and also mix the cytoplasm to enhance the biomass diffusion and adsorb much more kinds of biomolecules on the SERS hotspots. The screenshots of a rotating MNM-SP in a single cell at different time intervals are shown in Figure 4(c). We could even observe the fluctuation of the cell membrane due to the rotation of the rod-like MNM-SP in the cell as highlighted by the red circle (dotted line) in Movie S5. We studied the rotating speed in a cell with different frequencies of the magnetic field (20 mT) (Figure 4(d)). Compared with the situation in DI water, the rotating speed in cells has a similar increasing trend with the rotating frequency of the magnetic field increasing up to 7 Hz, indicating effective magnetic activation of the MNM-SPs inside the cells. Furthermore, the rotating speed of the MNM-SP reached the maximum when the frequency of the magnetic field was 7 Hz that was used for the followed experiments.

After MNM-SP rotating with different times and speed, we captured the intracellular Raman spectra at the site of the MNM-SP (Figure S10). Much more characteristic Raman peaks containing additional information of biomolecules inside the cytoplasm appeared with the rotating time and speed increasing, which support our hypothesis that the actively rotating MNM-SPs can enhance the chance of contacts between biomolecules and SERS hotspots. A series of characteristic Raman peaks corresponding to different bonds from various biomolecules in living cells were captured (see Table 1).

Two typical Raman spectra before and after rotation of the MNM-SPs are presented in Figures 4(e) and 4(f), respectively. Characteristic peaks that come from amino acids, lipids, and carbohydrates ranging from 200 to 1600 cm^−1^ were captured [75]. The intensity of the characteristic peaks was enhanced with apparently improved signal-to-noise ratio (Figure 4(f)) compared to that without rotation (Figure 4(e)). Then, without MNM-SP rotary mixing, Raman peaks mainly distribute from 400 to 1200 cm^−1^ which is similar to previously reported results by conventional passive SERS probes [68, 75]. However, extra Raman peaks ranging from 1600 to 2600 cm^−1^ after rotary mixing were captured (Figure 4(f)), e.g., Raman peaks from amide I, suggesting that the actively self-rotating MNM-SPs could probably escape the endosome trapping and induce micromixing inside the cytoplasm or even break some subcellular organelles, to detect much more Raman information from inside the cell.

## 3. Discussion

In summary, we have presented a “rod-like” magnetic nanomotor-based active SERS probe (MNM-SPs). The current work endows conventional SERS probes with “intelligence” by the controllable motion behavior. The MNM-SPs can navigate on-demand to approach targeted sensing site at a small scale. In virtue of magnetically driven self-rotation, the MNM-SPs can enhance SERS sensing capability by improving the contact chance between SERS probes and analytes. Furthermore, we for the first time demonstrated the recovery and reuse of a single SERS probe by controlled self-cleaning process, which might be useful for future biochemical sensing at the micro-/nanoscale. To better demonstrate the practical significance of the MNM-SPs, we demonstrated on-demand targeting towards a single cell by an MNM-SP which further entered into the cell by magnetic force-assisted endocytosis. By rotating in an intracellular environment, the MNM-SPs revealed much more Raman peaks with enhanced signal-to-noise ratio compared to previously reported passive SERS probes, which can be attributed to the micromixing effect of the MNM-SPs inside the cells. The current work, integration of magnetically activated micro-/nanomotors with ultrasensitive SERS technology, can inspire future exploration on the design and construction of controllable micro-/nanomotor equipped with other advanced functional moieties to extend the applications of micro-/nanomotors. Current MNM-SPs can actively approach and contact with targeted cell but still cannot directly overcome the natural barriers of the cell membrane. In future work, nanomotors with other geometries should be explored in order to overcome biological barriers such as the cell membrane. In addition, further analysis on the obtained intracellular Raman signals, in particular the extra signals probably from the cytoplasm after active rotation, should be carried out in order to better understand the biological significance of MNM-SP-based intracellular SERS sensing. For further advancement of the MNM-SPs' sensing capability, Raman reporter molecules that are responsive to specific chemical species or signals like H_2_O_2_ or pH can be modified onto the MNM-SP, to achieve a remotely controllable nanoprobe for on-demand sensing of specifically targeted analytes.

## 4. Materials and Methods

### 4.1. Materials and Instruments

Tethaethylorthosilicate (TEOS, 99%), ammonia (25%), ethanol (EtOH, >99%), isopropanol (IPA, 99.5%), ferric chloride (analytical grade), silver nitrate (analytical grade), and polyvinylpyrrolidone (PVP, 24 kDa) are commercially purchased and used as received. Scanning electron microscopy (SEM) images were captured by a field emission SEM (FESEM) S4700 at 15 kV. Transmission electron microscopy (TEM) images were captured at 120 kV by Tecnai G2 Spirit. Optical videos were captured by a Leica (DMi8) inverted optical microscope with 40x air objective. The Raman measurement was carried out by Horiba (Horiba LabRAM HR Evolution) equipped with 514 nm laser.

### 4.2. Fabrication of the Fe_3_O_4_ Nanoparticles

The Fe_3_O_4_ nanoparticles were synthesized by hydrothermal reaction [68, 69]. First, FeCl_3_·6H_2_O (0.675 g) was dissolved in ethylene glycol (35 mL) by constant ultrasonic treatment. Then, ammonium acetate (1.925 g) was added into the previous mixture and dissolved by magnetic stirring for 30 min. Finally, the mixture was transferred to a reaction kettle and heated at 200°C for 12 h. After cooling to room temperature, the synthesized Fe_3_O_4_ nanoparticles were collected by centrifugation (8000 r/min) for 5 min and washed with ethanol for 3 times. The collected Fe_3_O_4_ nanoparticles were dried in air for further experiment.

### 4.3. Fabrication of the Fe_3_O_4_@SiO_2_ Nanorods

Silica shell was coated on the Fe_3_O_4_ surface. The above dried Fe_3_O_4_ nanoparticles (4 mg) were added into a mixture of DI water (5 mL) and isopropanol (25 mL) and sonicated for 30 min. Then, ammonia (0.5 mL) was added to the mixture which was shaken for 10 min and an extramagnetic field was applied on the reaction system. Then, TOES (40 *μ*L) was added into the mixture and the reaction was kept at room temperature for 6 h with shaking. The silica-coated Fe_3_O_4_ nanorods were collected by centrifugation (8000 r/min for 5 min) and washed by ethanol.

### 4.4. Fabrication of the Fe_3_O_4_@SiO_2_@Ag

Silver nanoparticles were grown on a silica shell by silver mirror reaction. Typically, PVP (1 g) was dissolved in ethanol (13 mL) by ultrasonic treatment. Then, silver nitrate (300 *μ*L) was added into DI water and stirred by a magnetic stirrer for 30 min. The previous mixture was added into the PVP solution with Fe_3_O_4_@SiO_2_ and shaken for 5 min. Finally, the mixture was transferred into a reaction kettle and heated at 160°C for 6 h. After cooling to room temperature, the Fe_3_O_4_@SiO_2_@Ag was collected by centrifugation (7000 r/min for 5 min) and washed by DI water for 3 times.

### 4.5. SERS Signal Detection of Rhodamine 6G (R6G) and Crystal Violet (CV)

SERS spectra of different concentrations of R6G (10^−7^~10^−10^ M) and CV (10^−5^~10^−9^ M) were detected by adding the MNM-SPs into the solution of R6G (or CV), and the Raman spectra were collected by using a confocal Raman spectrometer (Horiba LabRAM HR Evolution) with 514 nm laser with an integration time of 30 s.

### 4.6. Microchannel Design and Print

SOLIDWORKS was used for designing the microchannel with three circular tanks connected with a twisting channel, which was fabricated by 3D printing (SPSS-450).

### 4.7. Optical Video Recording

The motion of the nanomotors was observed by using a Leica optical inverted microscope equipped with a 40x air objective. The nanomotors were placed in a petri dish filled with DI water. A glass slide was used to cover the petri dish in order to minimize the convection of the solution. Videos were recorded by a CCD camera at a frame rate of about 25 fps.

### 4.8. Cell Membrane and Nucleus Staining and CLSM Observation

First, cells were washed twice with preheated PBS (pH = 7.4, 37°C). Then, the cells were fixed in 4% paraformaldehyde for 15 minutes and washed 3 times with PBS. After that, cells were incubated with red fluorescent probe (1,1′-dioctadecyl-3,3,3′,3′-tetramethylindocy-cloanine perchlorate, Dil) solution at 37°C for 10 minutes, and then washed with PBS 3 times. Finally, the cells were incubated in 0.03 *μ*g/mL DAPI for 5 minutes and washed with PBS 3 times. After staining, the cells were sealed on a glass slide for observation by CLSM (Nikon A1) in blue, green, and red channels.

### 4.9. Magnetic Control System

The home-made magnetic field generator consisted of a CCD camera, microscope (Lecia DMi8), function generator (FY8300S), power amplifier (HSLFSun GLY-FP1000), and magnetic field generator (Figure S11). Different functions obtained from a function generator were amplified to drive the magnetic field generator to produce the required magnetic field. The magnetic control system was coupled with microscopy for observing and tracking MNM-SPs, and movement videos were recorded by a CCD camera at a frame rate of about 25 fps.

## Figures and Tables

**Table 1:** Raman peak assignments for the intracellular SERS signals captured with MNM-SPs in the HepG-2 cell [71, 78–80].

Raman shift (cm^−1^)	Assignment	Raman shift (cm^−1^)	Assignment
564	C-cl stretching	2096	C ≡ C stretching
785	Cytosine, uracil (ring, stretching)	2237	N=C=O stretching
1086	C-O stretching	2410	C ≡ CH stretching
1102	PO_2_- stretching (symmetric)	2592	S-H stretching
1323	Guanine	2933	CH_3_ and CH_2_ stretching
1441	CH_2_ deformation	3066	(C=CH-H) stretching
1650-1680	Amide I		

## Supplementary Materials

Supplementary 1 Figure S1: schematic illustration of (a) Fe_3_O_4_ nanoparticles with different orientations of magnetic moment, (b) aligned into chain within external magnetic field, and (c) then coated with silica layer to fix the “rod-like” structure. Figure S2: typical SEM images of the “rod-like” core-shell structures of silica-coated Fe_3_O_4_ nanoparticles. Figure S3: the length of the prepared nanorods coated with a thin layer of silica with different applied extramagnetic intensity with the same reaction time (6 h). Figure S4: (a) and (b) are the intensity of the CV and R6G in 1173 cm^−1^ and 1307 cm^−1^, respectively, with the different concentrations. The inserted pictures are chemical formulae of dye molecules. Error bars indicate standard deviation (*N* = 10). Figure S5: schematic illustration of a rotating mechanism of the “rod-like” nanomotor in the rotary magnetic field. Orange arrow is the orientation of the magnetic field and *θ* is the angle between magnetic moment of “rod-like” nanomotor and the magnetic field. Figure S6: average rotating speed of the MNM-SPs in silica oil (Cst = 500) under the rotary magnetic field with different frequencies (magnetic strength is 20 mT). Error bars indicate standard deviation (*N* = 5). Figure S7: scheme (a) and 3D print (b) of a microchannel with three tanks connected with a twisting channel. Figure S8: (a), (b), and (c) are the Raman spectra of analyte 1(CV) sensing, CV-contaminated probes, and analyte 2(R6G) sensing, respectively, with different standing times. (d), (e), and (f) are the intensity variation of the prominent peaks of CV and R6G in (a), (b), and (c), respectively. Error bars indicate standard deviation (*N* = 5). Figure S9: fluorescent spectrum of fluorescein isothiocyanate (FITC) and UV-Vis absorption spectrum of Fe_3_O_4_, Fe_3_O_4_@SiO_2_, Fe_3_O_4_@SiO_2_&FITC, respectively. Figure S10: SERS spectra from the site of MNM-SPs within an intracellular environment with (a) different rotating times (frequency of magnetic field is 7 Hz) and (b) different frequencies of magnetic field (rotating time is 15 min). Figure S11: the parts of a home-made magnetic field generator are as follows: (a) adjustable function generator, (b) signal amplifier, (c) fluorescent microscope assembled with magnetic coil setup, and (d) detailed view of magnetic field coils. Table S1: peak assignments for crystal violet (CV) SERS spectra. Table S2: peak assignments for rhodamine 6G (R6G) SERS spectra.

Supplementary 2 Movie S1: MNM-SPs navigating in deionized water with gradient magnetic field actuating.

Supplementary 3 Movie S2: MNM-SPs rotating in deionized water with different frequencies of rotary magnetic field.

Supplementary 4 Movie S3: MNM-SPs approaching a targeted cell with the assistance of a gradient magnetic field.

Supplementary 5 Movie S4: MNM-SPs endocytosed into a targeted cell imaged by confocal laser scanning microscope.

Supplementary 6 Movie S5: MNM-SPs rotating in a cell with different frequencies of rotary magnetic field.

## References

[1] Wang H., Pumera M. (2015). Fabrication of micro/nanoscale motors. *Chemical Reviews*.

[2] Wang J. (2013). *Nanomachines: Fundamentals and Applications*.

[3] Sánchez S., Soler L., Katuri J. (2015). Chemically powered micro- and nanomotors. *Angewandte Chemie International Edition*.

[4] Ma X., Jang S., Popescu M. N. (2016). Reversed janus micro/nanomotors with internal chemical engine. *ACS Nano*.

[5] Ma X., Hortelão A. C., Patiño T., Sánchez S. (2016). Enzyme catalysis to power micro/nanomachines. *ACS Nano*.

[6] Zhu H., Nawar S., Werner J. G. (2019). Hydrogel micromotors with catalyst-containing liquid core and shell. *Journal of Physics: Condensed Matter*.

[7] Naeem S., Naeem F., Manjare M. (2019). Tubular catalytic micromotors in transition from unidirectional bubble sequences to more complex bidirectional motion. *Applied Physics Letters*.

[8] Nourhani A., Karshalev E., Soto F., Wang J. (2020). Multigear bubble propulsion of transient micromotors. *Research*.

[9] Xu T., Gao W., Xu L. P., Zhang X., Wang S. (2017). Fuel-free synthetic micro-/nanomachines. *Advanced Materials*.

[10] Yu J., Wang B., Du X., Wang Q., Zhang L. (2018). Ultra-extensible ribbon-like magnetic microswarm. *Nature Communications*.

[11] Wang W., Li S., Mair L., Ahmed S., Huang T. J., Mallouk T. E. (2014). Acoustic propulsion of nanorod motors inside living cells. *Angewandte Chemie International Edition*.

[12] Xu L., Mou F., Gong H., Luo M., Guan J. (2017). Light-driven micro/nanomotors: from fundamentals to applications. *Chemical Society Reviews*.

[13] Liang Z., Fan D. (2018). Visible light–gated reconfigurable rotary actuation of electric nanomotors. *Science Advances*.

[14] Medina-Sánchez M., Schmidt O. G. (2017). Medical microbots need better imaging and control. *Nature*.

[15] Magdanz V., Sanchez S., Schmidt O. G. (2013). Development of a sperm-flagella driven micro-bio-robot. *Advanced Materials*.

[16] Xu H., Medina-Sánchez M., Magdanz V., Schwarz L., Hebenstreit F., Schmidt O. G. (2018). Sperm-hybrid micromotor for targeted drug delivery. *ACS Nano*.

[17] Baraban L., Makarov D., Streubel R. (2012). Catalytic Janus motors on microfluidic chip: deterministic motion for targeted cargo delivery. *ACS Nano*.

[18] Garcia-Gradilla V., Orozco J., Sattayasamitsathit S. (2013). Functionalized ultrasound-propelled magnetically guided nanomotors: toward practical biomedical applications. *ACS Nano*.

[19] Xu D., Wang Y., Liang C., You Y., Sanchez S., Ma X. (2019). Self-propelled micro/nanomotors for on-demand biomedical cargo transportation. *Small*.

[20] Luo M., Feng Y., Wang T., Guan J. (2018). Micro-/nanorobots at work in active drug delivery. *Advanced Functional Materials*.

[21] Leong T. G., Randall C. L., Benson B. R., Bassik N., Stern G. M., Gracias D. H. (2009). Tetherless thermobiochemically actuated microgrippers. *Proceedings of the National Academy of Sciences*.

[22] Srivastava S. K., Medina-Sánchez M., Koch B., Schmidt O. G. (2016). Medibots: dual-action biogenic microdaggers for single-cell surgery and drug release. *Advanced Materials*.

[23] Gao W., Wang J. (2014). The environmental impact of micro/nanomachines: a review. *ACS Nano*.

[24] Zarei M., Zarei M. (2018). Self-propelled micro/nanomotors for sensing and environmental remediation. *Small*.

[25] Chang J., Zhang L., Wang P. (2018). Intelligent environmental nanomaterials. *Environmental Science: Nano*.

[26] Parmar J., Vilela D., Villa K., Wang J., Sánchez S. (2018). Micro- and nanomotors as active environmental microcleaners and sensors. *Journal of the American Chemical Society*.

[27] Jurado-Sánchez B., Escarpa A. (2017). Janus micromotors for electrochemical sensing and biosensing applications: a review. *Electroanalysis*.

[28] Kagan D., Calvo-Marzal P., Balasubramanian S. (2009). Chemical sensing based on catalytic nanomotors: motion-based detection of trace silver. *Journal of the American Chemical Society*.

[29] Bunea A.-I., Pavel I.-A., David S., Gáspár S. (2015). Sensing based on the motion of enzyme-modified nanorods. *Biosensors and Bioelectronics*.

[30] Orozco J., García-Gradilla V., D’Agostino M., Gao W., Cortes A., Wang J. (2012). Artificial enzyme-powered microfish for water-quality testing. *ACS Nano*.

[31] Wang Y., Zhou C., Wang W. (2018). Photocatalytically powered matchlike nanomotor for light-guided active SERS sensing. *Angewandte Chemie International Edition*.

[32] Su Y., Ge Y., Liu L. (2016). Motion-based pH sensing based on the cartridge-case-like micromotor. *ACS Applied Materials & Interfaces*.

[33] Liu L., Dong Y., Sun Y. (2016). Motion-based pH sensing using spindle-like micromotors. *Nano Research*.

[34] Patino T., Porchetta A., Jannasch A. (2019). Self-sensing enzyme-powered micromotors equipped with pH-responsive DNA nanoswitches. *Nano Letters*.

[35] Kong L., Rohaizad N., Nasir M. Z. M., Guan J., Pumera M. (2019). Micromotor-assisted human serum glucose biosensing. *Analytical Chemistry*.

[36] Wu J., Balasubramanian S., Kagan D., Manesh K. M., Campuzano S., Wang J. (2010). Motion-based DNA detection using catalytic nanomotors. *Nature Communications*.

[37] Van Nguyen K., Minteer S. D. (2015). DNA-functionalized Pt nanoparticles as catalysts for chemically powered micromotors: toward signal-on motion-based DNA biosensor. *Chemical Communications*.

[38] Draz M. S., Lakshminaraasimulu N. K., Krishnakumar S. (2018). Motion-based immunological detection of Zika virus using Pt-nanomotors and a cellphone. *ACS Nano*.

[39] Esteban-Fernández de Ávila B., Martín A., Soto F. (2015). Single cell real-time miRNAs sensing based on nanomotors. *ACS Nano*.

[40] Zhang Y., Zhang L., Yang L. (2019). Real-time tracking of fluorescent magnetic spore–based microrobots for remote detection ofC. difftoxins. *Science Advances*.

[41] Jurado-Sánchez B., Escarpa A., Wang J. (2015). Lighting up micromotors with quantum dots for smart chemical sensing. *Chemical Communications*.

[42] Pacheco M., Jurado-Sánchez B., Escarpa A. (2018). Sensitive monitoring of enterobacterial contamination of food using self-propelled Janus microsensors. *Analytical Chemistry*.

[43] Jurado-Sánchez B., Pacheco M., Rojo J., Escarpa A. (2017). Magnetocatalytic graphene quantum dots Janus micromotors for bacterial endotoxin detection. *Angewandte Chemie International Edition*.

[44] Molinero-Fernández Á., Moreno-Guzmán M., López M. Á., Escarpa A. (2017). Biosensing strategy for simultaneous and accurate quantitative analysis of mycotoxins in food samples using unmodified graphene micromotors. *Analytical Chemistry*.

[45] Singh V. V., Kaufmann K., Orozco J. (2015). Micromotor-based on–off fluorescence detection of sarin and soman simulants. *Chemical Communications*.

[46] Ding S.-Y., You E.-M., Tian Z.-Q., Moskovits M. (2017). Electromagnetic theories of surface-enhanced Raman spectroscopy. *Chemical Society Reviews*.

[47] Zong C., Xu M., Xu L.-J. (2018). Surface-enhanced Raman spectroscopy for bioanalysis: reliability and challenges. *Chemical Reviews*.

[48] Zhan C., Chen X.-J., Yi J., Li J.-F., Wu D.-Y., Tian Z.-Q. (2018). From plasmon-enhanced molecular spectroscopy to plasmon-mediated chemical reactions. *Nature Reviews Chemistry*.

[49] Procházka M. (2016). *"Surface-Enhanced Raman Spectroscopy", Biological and Medical Physics, Biomedical Engineering*.

[50] Hudson S. D., Chumanov G. (2009). Bioanalytical applications of SERS (surface-enhanced Raman spectroscopy). *Analytical and Bioanalytical Chemistry*.

[51] Yin Y., Qiu T., Ma L. (2012). Exploring rolled-up Au–Ag bimetallic microtubes for surface-enhanced Raman scattering sensor. *The Journal of Physical Chemistry C*.

[52] Su X., Sutarlie L., Loh X. J. (2020). Sensors, biosensors, and analytical technologies for aquaculture water quality. *Research*.

[53] Zeng F., Duan W., Zhu B. (2018). Based versatile surface-enhanced Raman spectroscopy chip with smartphone-based Raman analyzer for point-of-care application. *Analytical Chemistry*.

[54] Zhang K., Wang Y., Wu M., Liu Y., Shi D., Liu B. (2018). On-demand quantitative SERS bioassays facilitated by surface-tethered ratiometric probes. *Chemical Science*.

[55] Zeng F., Xu D., Zhan C. (2018). Surfactant-free synthesis of graphene oxide coated silver nanoparticles for Sers biosensing and intracellular drug delivery. *ACS Applied Nano Materials*.

[56] Jiang X., Tan Z., Lin L. (2018). Surface-enhanced Raman nanoprobes with embedded standards for quantitative cholesterol detection. *Small Methods*.

[57] Qiu Y., Zhang Y., Li M. (2018). Intraoperative detection and eradication of residual microtumors with gap-enhanced raman tags. *ACS Nano*.

[58] Ren S., Wang J., Song C. (2019). Single-step organization of plasmonic gold metamaterials with self-assembled DNA nanostructures. *Research*.

[59] Novotný F., Plutnar J., Pumera M. (2019). Plasmonic self-propelled nanomotors for explosives detection via solution-based surface enhanced Raman scattering. *Advanced Functional Materials*.

[60] Han D., Fang Y., Du D., Huang G., Qiu T., Mei Y. (2016). Automatic molecular collection and detection by using fuel-powered microengines. *Nanoscale*.

[61] Chen X.-Z., Hoop M., Mushtaq F. (2017). Recent developments in magnetically driven micro- and nanorobots. *Applied Materials Today*.

[62] Chen X. Z., Jang B., Ahmed D. (2018). Small-scale machines driven by external power sources. *Advanced Materials*.

[63] Erb R. M., Martin J. J., Soheilian R., Pan C., Barber J. R. (2016). Actuating soft matter with magnetic torque. *Advanced Functional Materials*.

[64] Peyer K. E., Zhang L., Nelson B. J. (2013). Bio-inspired magnetic swimming microrobots for biomedical applications. *Nanoscale*.

[65] Wu Z., Troll J., Jeong H.-H. (2018). A swarm of slippery micropropellers penetrates the vitreous body of the eye. *Science Advances*.

[66] Xin C., Yang L., Li J. (2019). Conical hollow microhelices with superior swimming capabilities for targeted cargo delivery. *Advanced Materials*.

[67] Yu J., Jin D., Chan K.-F., Wang Q., Yuan K., Zhang L. (2019). Active generation and magnetic actuation of microrobotic swarms in bio-fluids. *Nature Communications*.

[68] Xu D., Xie R., Xu T. (2016). Combination therapeutics of doxorubicin with Fe_3_O_4_@ chitosan@ phytic acid nanoparticles for multi-responsive drug delivery. *RSC Advances*.

[69] Yang X., Jiang W., Liu L. (2012). One-step hydrothermal synthesis of highly water-soluble secondary structural Fe_3_O_4_ nanoparticles. *Journal of Magnetism and Magnetic Materials*.

[70] Ma H., Tang K., Luo W. (2017). Photonic nanorods with magnetic responsiveness regulated by lattice defects. *Nanoscale*.

[71] El-Said W. A., Kim T.-H., Kim H., Choi J.-W. (2010). Detection of effect of chemotherapeutic agents to cancer cells on gold nanoflower patterned substrate using surface-enhanced Raman scattering and cyclic voltammetry. *Biosensors and Bioelectronics*.

[72] Liu R. M., Kang Y. P., Zi X. F., Feng M. J., Cheng M., Si M. Z. (2009). The ultratrace detection of crystal violet using surface enhanced Raman scattering on colloidal Ag nanoparticles prepared by electrolysis. *Chinese Chemical Letters*.

[73] Zhang C., Jiang S., Huo Y. (2015). SERS detection of R6G based on a novel graphene oxide/silver nanoparticles/silicon pyramid arrays structure. *Optics Express*.

[74] Mei Y., Huang G., Solovev A. A. (2008). Versatile approach for integrative and functionalized tubes by strain engineering of nanomembranes on polymers. *Advanced Materials*.

[75] Ureña E. B., Mei Y., Coric E., Makarov D., Albrecht M., Schmidt O. G. (2009). Fabrication of ferromagnetic rolled-up microtubes for magnetic sensors on fluids. *Journal of Physics D: Applied Physics*.

[76] Sykes E. A., Chen J., Zheng G., Chan W. C. (2014). Investigating the impact of nanoparticle size on active and passive tumor targeting efficiency. *ACS Nano*.

[77] Sawetzki T., Rahmouni S., Bechinger C., Marr D. W. (2008). In situ assembly of linked geometrically coupled microdevices. *Proceedings of the National Academy of Sciences*.

[78] Huang W. E., Griffiths R. I., Thompson I. P., Bailey M. J., Whiteley A. S. (2004). Raman microscopic analysis of single microbial cells. *Analytical Chemistry*.

[79] Maquelin K., Kirschner C., Choo-Smith L.-P. (2002). Identification of medically relevant microorganisms by vibrational spectroscopy. *Journal of Microbiological Methods*.

[80] Edwards H. (2006). Spectra–structure correlations in Raman spectroscopy. *Handbook of Vibrational Spectroscopy*.

