# Supplementary material for: Magnetic Nanomotor-Based Maneuverable SERS Probe
Source: Research (Wash D C). 2020 Jun 5;2020:7962024. doi: 10.34133/2020/7962024 (PMC7293755; doi:10.34133/2020/7962024)
Supplement: Supplementary 1 — Figure S1: schematic illustration of (a) Fe3O4 nanoparticles with different orientations of magnetic moment, (b) aligned into chain within external magnetic field, and (c) then coated with silica layer to fix the “rod-like” structure. Figure S2: typical SEM images of the “rod-like” core-shell structures of silica-coated Fe3O4 nanoparticles. Figure S3: the length of the prepared nanorods coated with a thin layer of silica with different applied extramagnetic intensity with the same reaction time (6 h). Figure S4: (a) and (b) are the intensity of the CV and R6G in 1173 cm−1 and 1307 cm−1, respectively, with the different concentrations. The inserted pictures are chemical formulae of dye molecules. Error bars indicate standard deviation (N = 10). Figure S5: schematic illustration of a rotating mechanism of the “rod-like” nanomotor in the rotary magnetic field. Orange arrow is the orientation of the magnetic field and θ is the angle between magnetic moment of “rod-like” nanomotor and the magnetic field. Figure S6: average rotating speed of the MNM-SPs in silica oil (Cst = 500) under the rotary magnetic field with different frequencies (magnetic strength is 20 mT). Error bars indicate standard deviation (N = 5). Figure S7: scheme (a) and 3D print (b) of a microchannel with three tanks connected with a twisting channel. Figure S8: (a), (b), and (c) are the Raman spectra of analyte 1(CV) sensing, CV-contaminated probes, and analyte 2(R6G) sensing, respectively, with different standing times. (d), (e), and (f) are the intensity variation of the prominent peaks of CV and R6G in (a), (b), and (c), respectively. Error bars indicate standard deviation (N = 5). Figure S9: fluorescent spectrum of fluorescein isothiocyanate (FITC) and UV-Vis absorption spectrum of Fe3O4, Fe3O4@SiO2, Fe3O4@SiO2&FITC, respectively. Figure S10: SERS spectra from the site of MNM-SPs within an intracellular environment with (a) different rotating times (frequency of magnetic field is 7 Hz) and (b) diff [file 7962024.f1.docx]

**Supplementary Information**

**Magnetic Nanomotor Based Maneuverable SERS Probe**

# Preparation and characterization


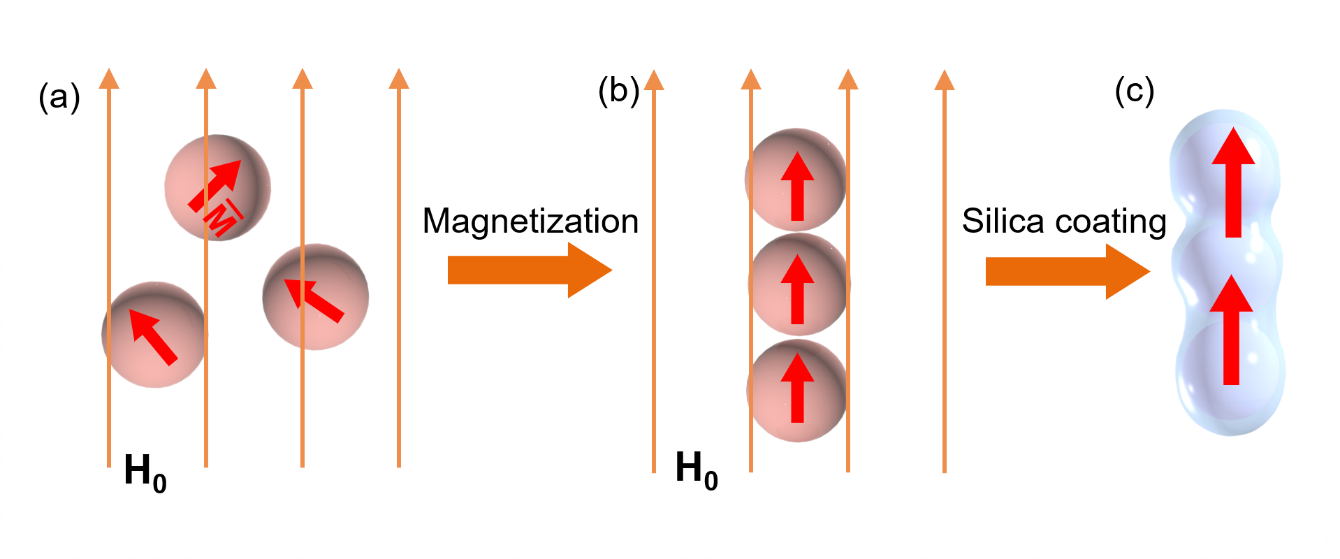


FIGURE S1: Schematic illustration of (a) Fe_3_O_4_ nanoparticles with different orientation of magnetic moment, (b) aligned into chain within external magnetic field, and (c) then coated with silica layer to fix the “rod-like” structure.


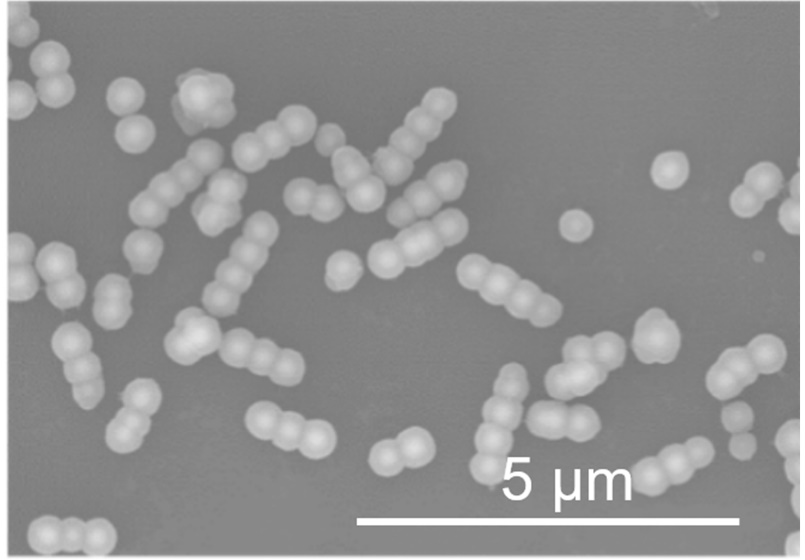


FIGURE S2: Typical SEM image of the “rod-like” core-shell structures of silica coated Fe_3_O_4_ nanoparticles.


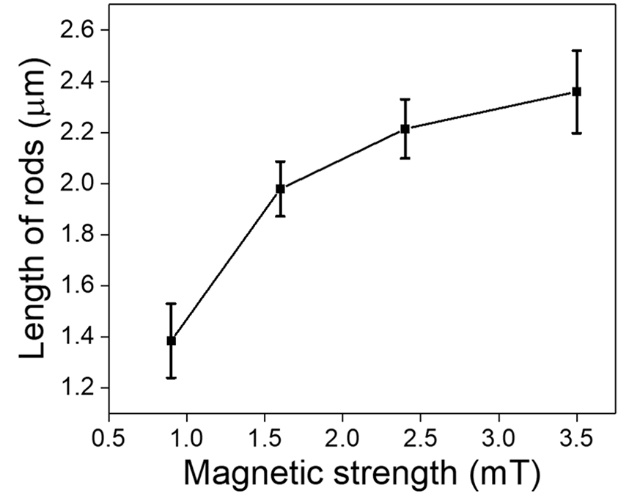


FIGURE S3: The length of the prepared silica coated magnetic nano-rods with different applied extra-magnetic intensity with the same reaction time (6 h). Error bars indicate one standard deviation (N=10).


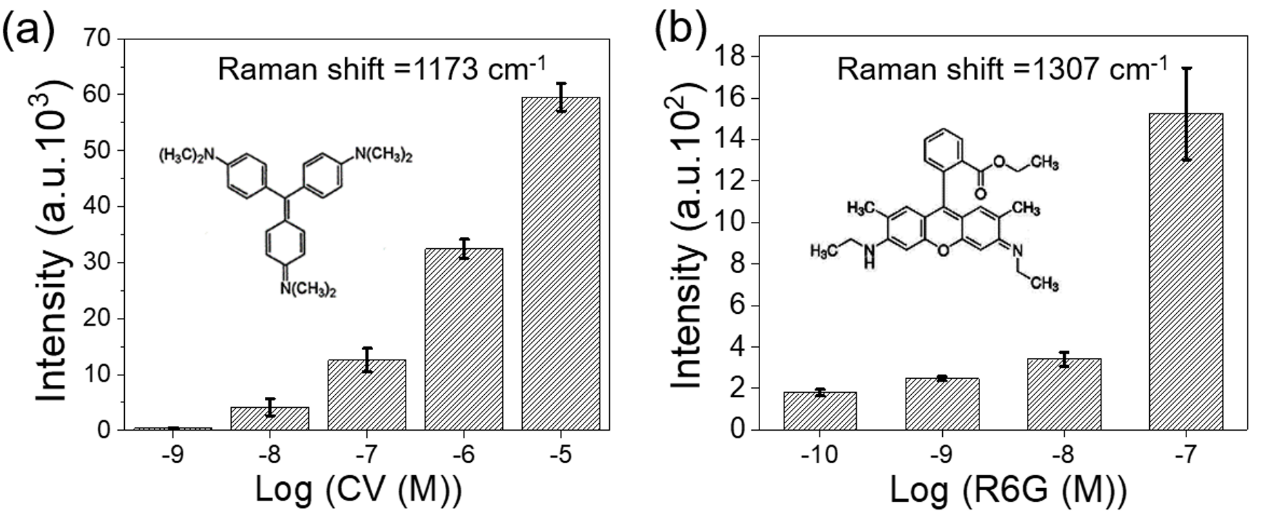


FIGURE S4: (a) and (b) are the intensity of the CV and R6G in 1173 cm^-1^ and 1307 cm^-1^ respectively with the different concentration. The inserted pictures are chemical formulae of the CV and R6G molecules. Error bars indicate standard deviation (N=10).

TABLE S1: Raman peak assignments for crystal violet (CV)[1-2]


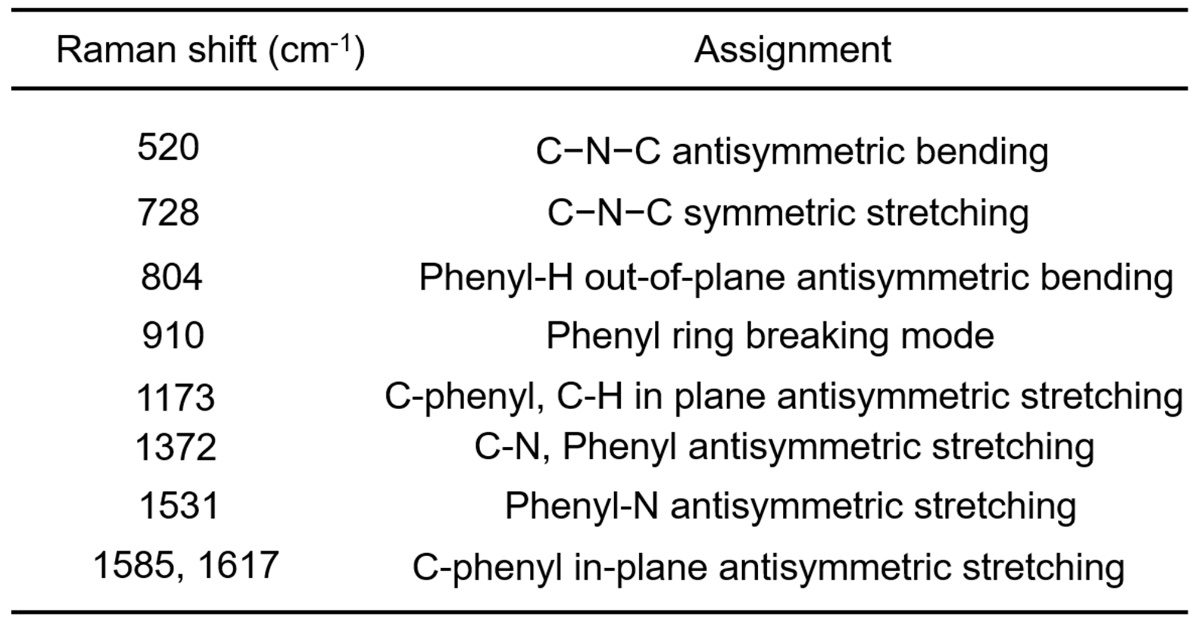


TABLE S2: Raman peak assignments for Rhodamine 6G (R6G)[3-4]


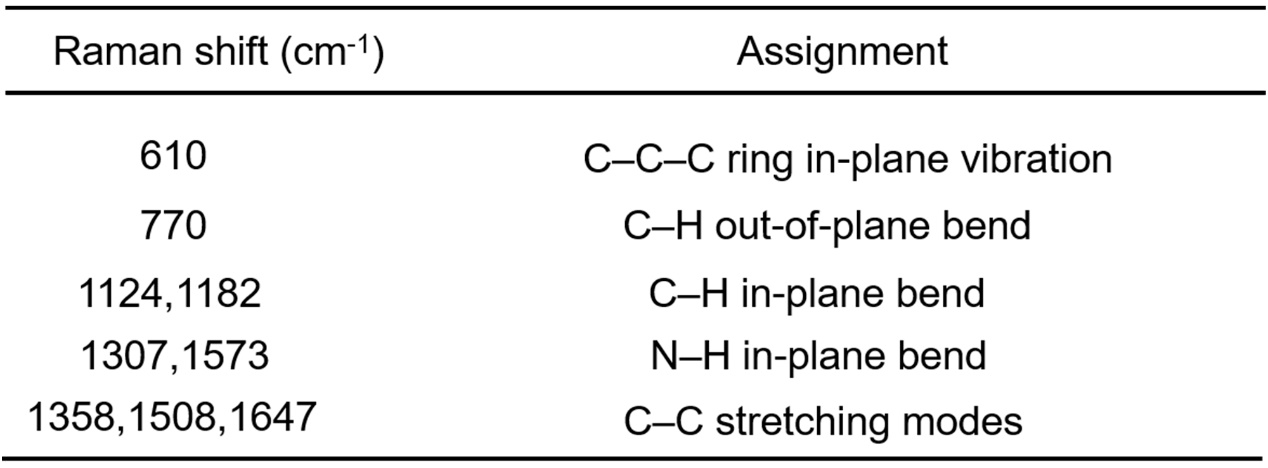


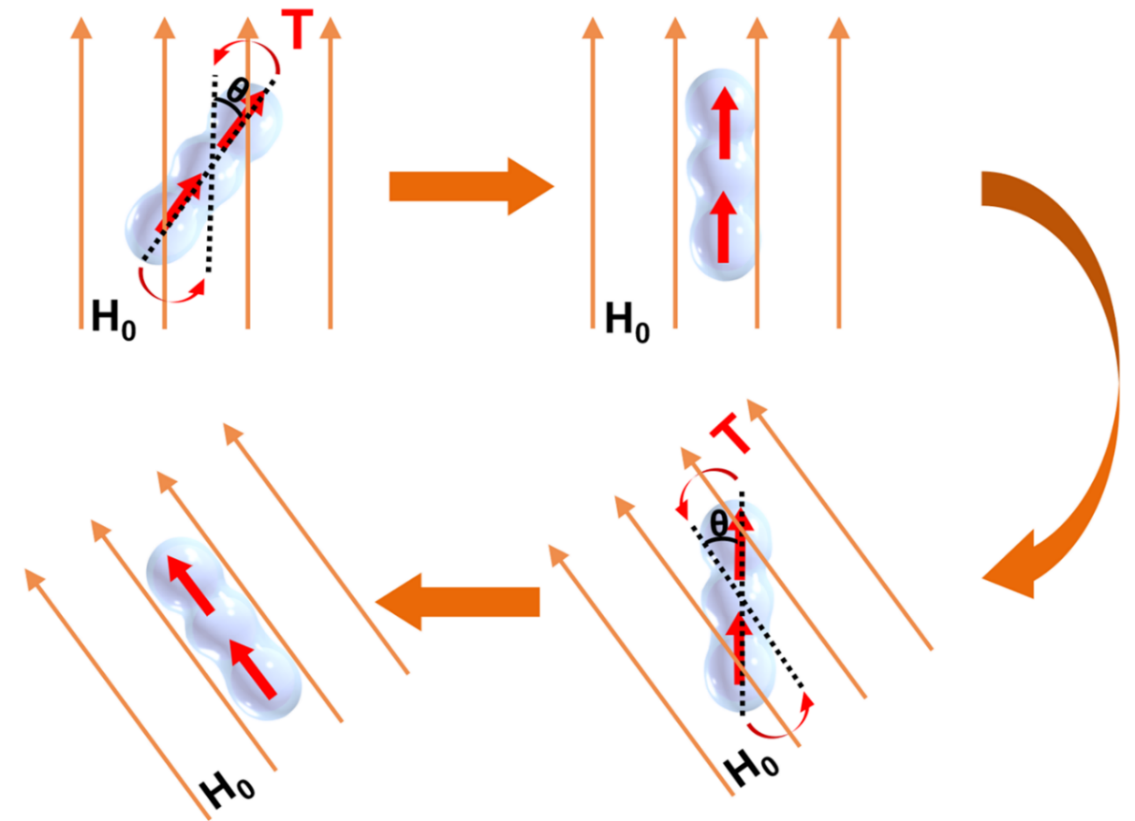


FIGURE S5: Schematic illustration of rotating mechanism of the “rod-like” nanomotor in rotary magnetic field. The orange arrow is orientation of the magnetic field and θ is the angle between magnetic moment of “rod-like” nanomotor and the magnetic field.


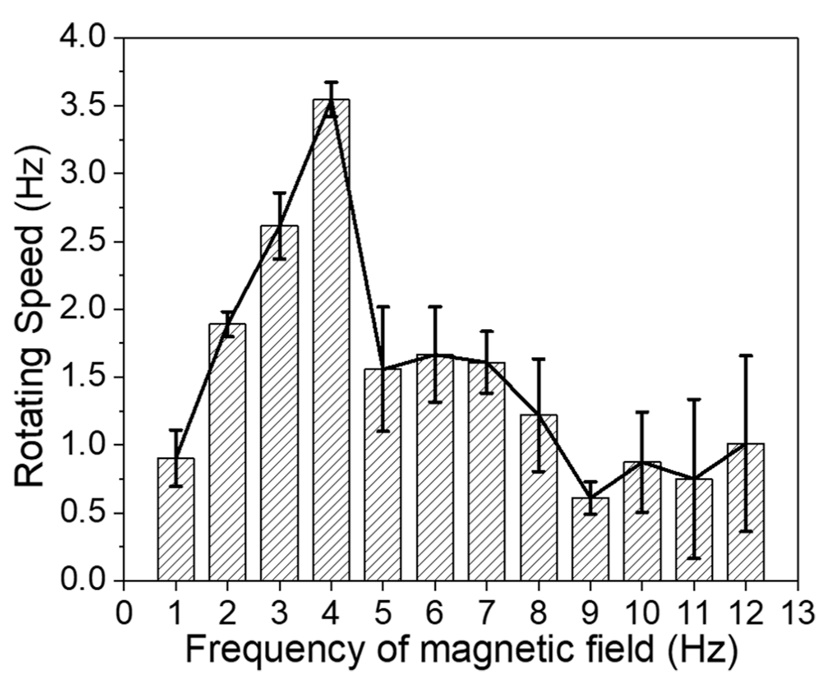


FIGURE S6: Average rotating speed of the MNM-SPs in silica oil (Cst=500) under rotary magnetic field with different frequency (magnetic strength is 20 mT). Error bars indicate standard deviation (N=5).


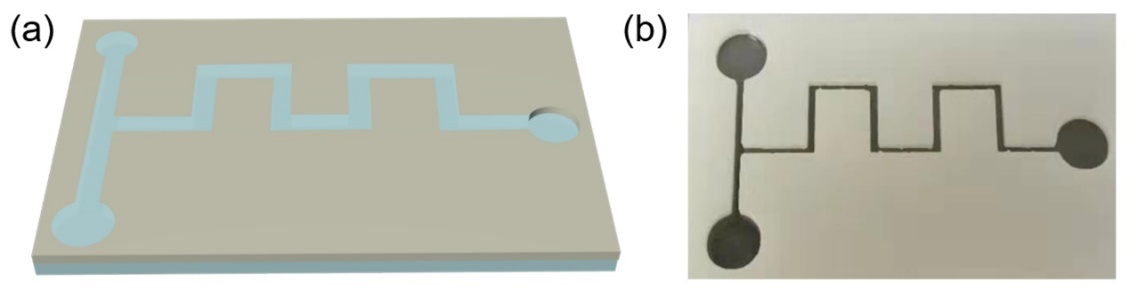


FIGURE S7: Scheme (a) and 3D print (b) of the microchannel with three tanks connected with a twisting channel.


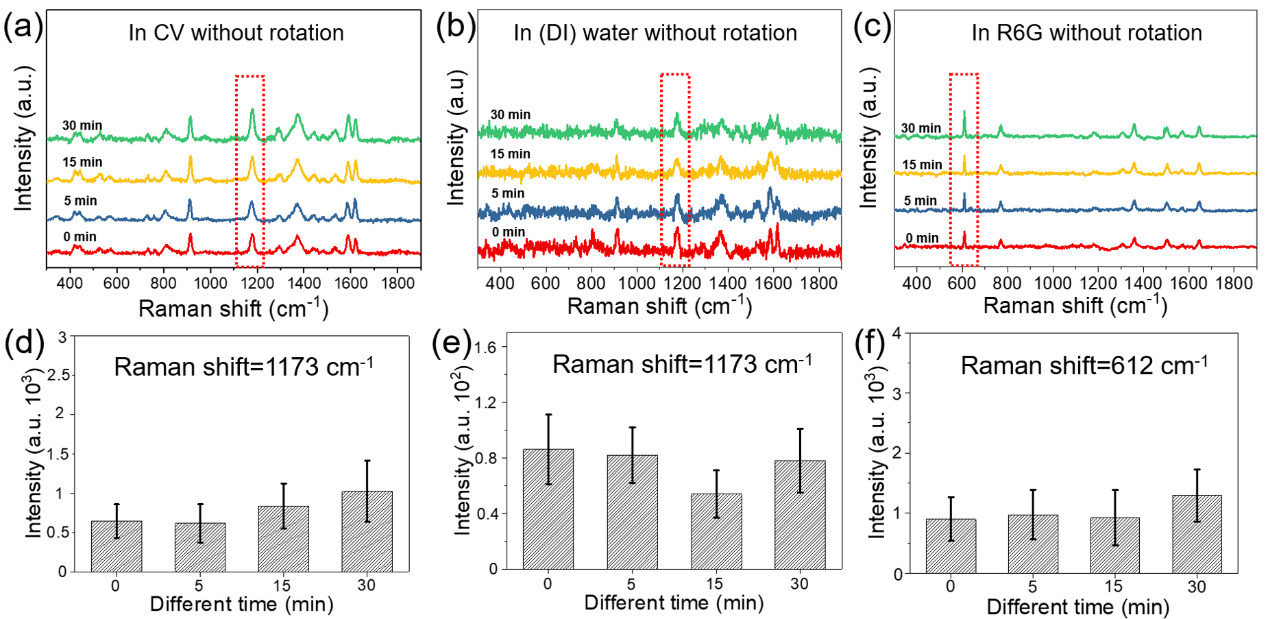


FIGURE S8: (a), (b), and (c) are the Raman spectra of the analyte 1(CV) sensing, CV contaminated probes and analyte 2(R6G) sensing, respectively, with different standing time. (d), (e), and (f) are the intensity variation of the prominent peaks of CV and R6G in (a), (b), and (c), respectively. Error bars indicate standard deviation (N=5).


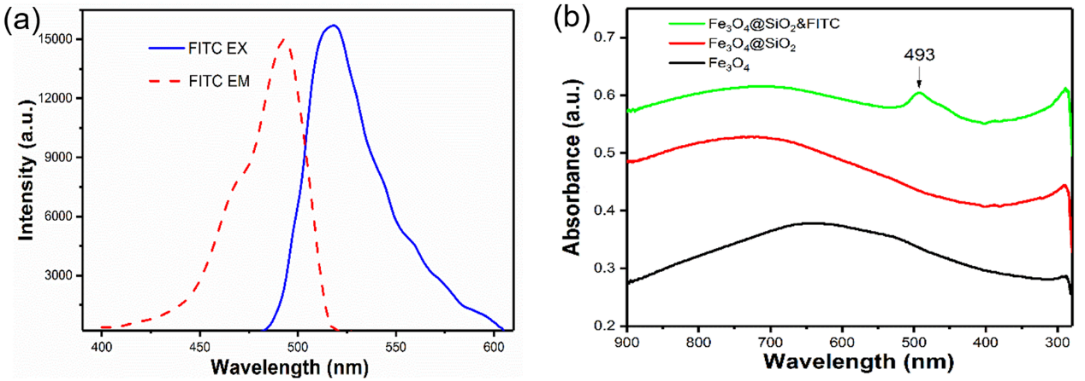


FIGURE S9: Fluorescent spectrum of fluorescein isothiocyanate (FITC) and UV-VIS absorption spectrum Fe_3_O_4_, Fe_3_O_4_@SiO_2_, Fe_3_O_4_@SiO_2_&FITC respectively.


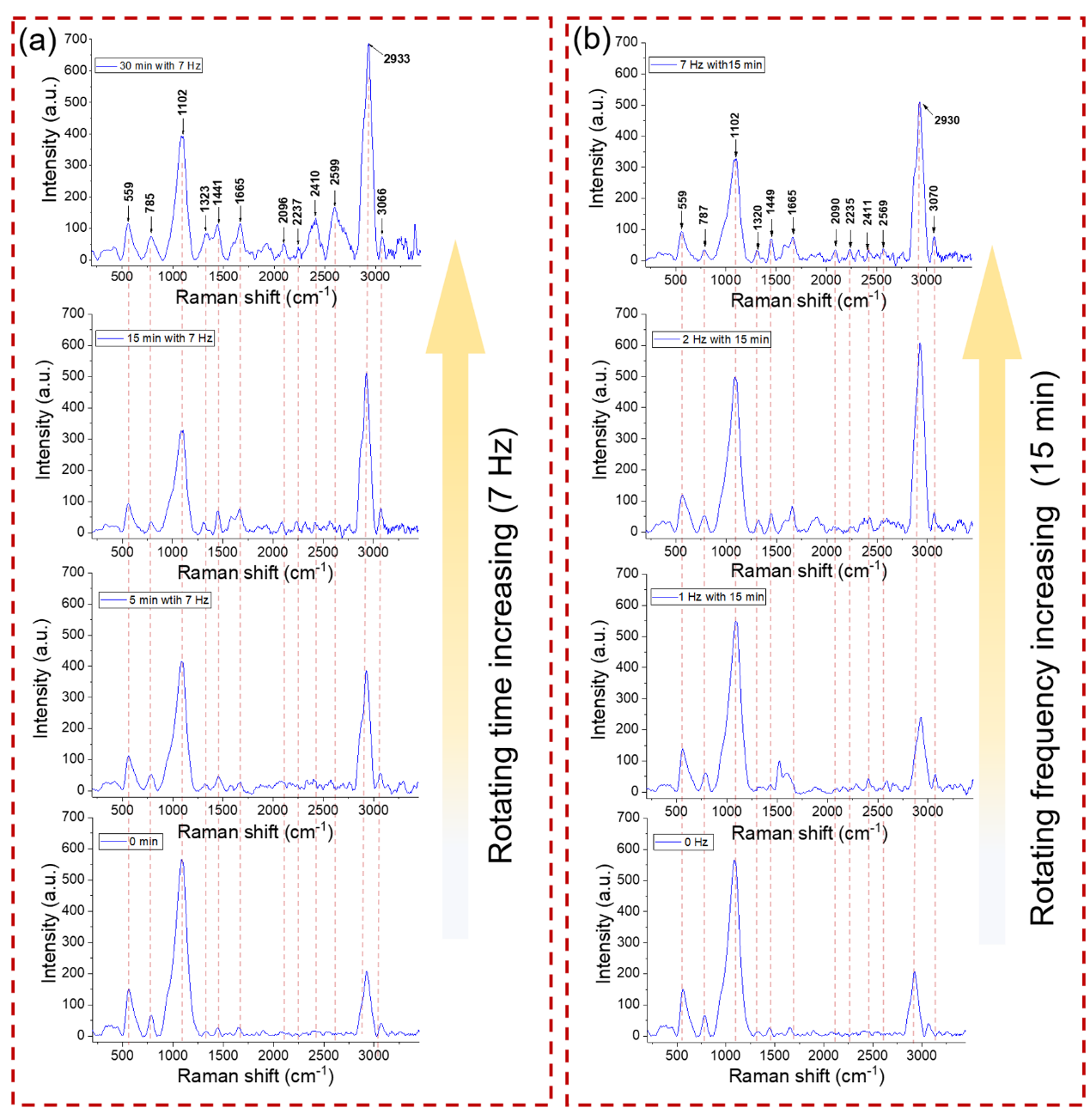


FIGURE S10: SERS spectra from the site of MNM-SPs within intracellular environment with (a) different rotating time (frequency of magnetic field is 7 Hz) and (b) different frequency of magnetic field (rotating time is 15 min).


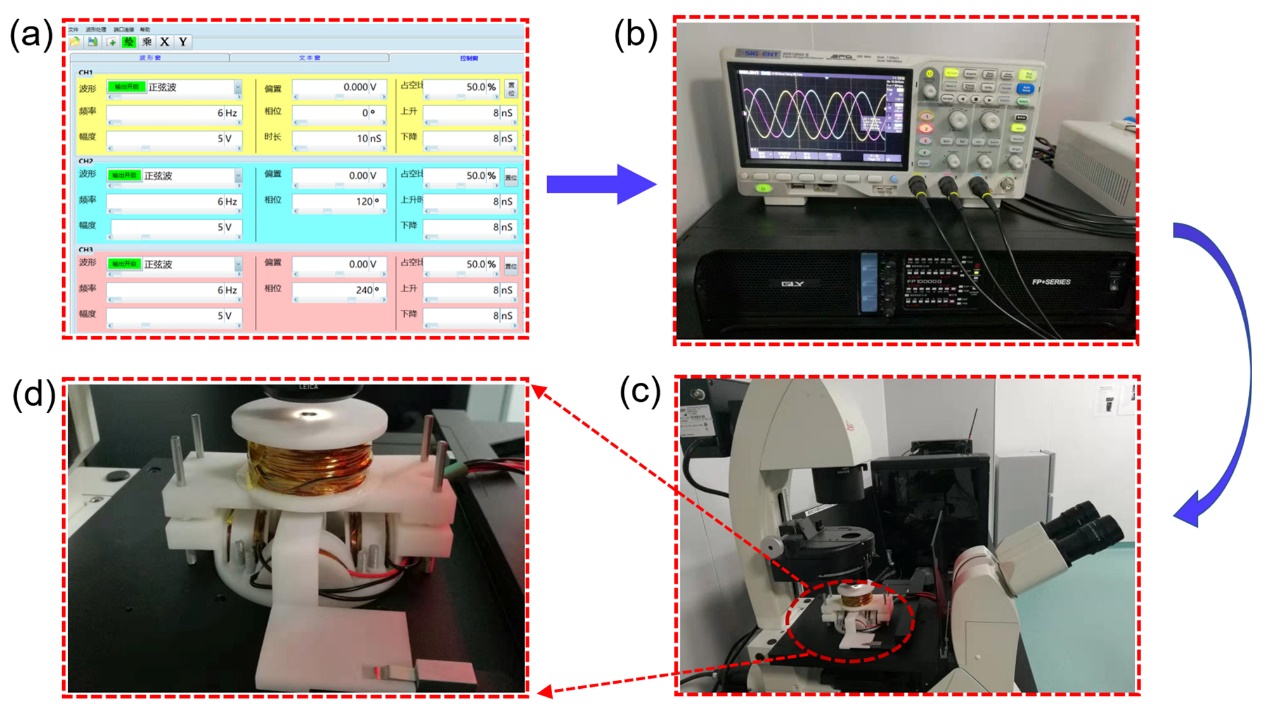


FIGURE S11: The constitutes of home-made magnetic field generator. (a) adjustable function generator, (b) signal amplifier, (c) fluorescent microscope assembled with magnetic coil setup, and (d) detailed view of magnetic field coils.

# Supplementary movies

Movie S1 MNM-SPs navigating in deionized water with gradient magnetic field actuating.

Movie S2 MNM-SPs rotating in deionized water with different frequency of rotary magnetic field.

Movie S3 MNM-SPs approaching to a targeted cell with the assistance of gradient magnetic field.

Movie S4 MNM-SPs endocytosed into a targeted cell imaged by confocal laser scanning microscope.

Movie S5 MNM-SPs rotating in a cell with different frequency of rotary magnetic field.

**3. References**

[1] R.M. Liu, Y.P. Kang, X.F. Zi, M.J. Feng, M. Cheng, M.Z. Si, "The ultratrace detection of crystal violet using surface enhanced Raman scattering on colloidal Ag nanoparticles prepared by electrolysis", *Chinese Chemical Letters,* vol. 20, no. 6, pp. 711-715, 2009.

[2] K. Lai, Y. Zhang, R. Du, F. Zhai, B.A. Rasco, Y. Huang, "Determination of chloramphenicol and crystal violet with surface enhanced Raman spectroscopy", *Sensing and Instrumentation for Food Quality and Safety,* vol. 5, no. 1, pp. 19-24, 2011.

[3] C. Zhang, S. Jiang, Y. Huo, A. Liu, S. Xu, X. Liu, Z. Sun, Y. Xu, Z. Li, B. Man, "SERS detection of R6G based on a novel graphene oxide/silver nanoparticles/silicon pyramid arrays structure", *Optics Express,* vol. 23, no. 19, pp. 24811-24821, 2015.

[4] R. Li, H. Li, S. Pan, K. Liu, S. Hu, L. Pan, Y. Guo, S. Wu, X. Li, J. Liu, "Surface-enhanced Raman scattering from rhodamine 6G on gold-coated self-organized silicon nanopyramidal array", *Journal of Materials Research,* vol. 28, no. 24, pp. 3401-3407, 2013.
